# Optical magnetism in planar metamaterial heterostructures

**DOI:** 10.1038/s41467-017-02589-8

**Published:** 2018-01-18

**Authors:** Georgia T. Papadakis, Dagny Fleischman, Artur Davoyan, Pochi Yeh, Harry A. Atwater

**Affiliations:** 1 Thomas J. Watson Laboratories of Applied Physics, California Institute of Technology, Pasadena, CA 91125 USA; 2 Kavli Nanoscience Institute, California Institute of Technology, Pasadena, CA 91125 USA; 3 Resnick Sustainability Institute, California Institute of Technology, Pasadena, CA 91125 USA; 4 Department of Electrical and Computer Engineering, University of Santa Barbara, Santa Barbara, CA 93106 USA

## Abstract

Harnessing artificial optical magnetism has previously required complex two- and three-dimensional structures, such as nanoparticle arrays and split-ring metamaterials. By contrast, planar structures, and in particular dielectric/metal multilayer metamaterials, have been generally considered non-magnetic. Although the hyperbolic and plasmonic properties of these systems have been extensively investigated, their assumed non-magnetic response limits their performance to transverse magnetic (TM) polarization. We propose and experimentally validate a mechanism for artificial magnetism in planar multilayer metamaterials. We also demonstrate that the magnetic properties of high-index dielectric/metal hyperbolic metamaterials can be anisotropic, leading to magnetic hyperbolic dispersion in certain frequency regimes. We show that such systems can support transverse electric polarized interface-bound waves, analogous to their TM counterparts, surface plasmon polaritons. Our results open a route for tailoring optical artificial magnetism in lithography-free layered systems and enable us to generalize the plasmonic and hyperbolic properties to encompass both linear polarizations.

## Abstract (web summary)

Most natural materials do not have a magnetic response at optical frequencies and inducing optical magnetism by metamaterials typically requires complex nanostructures. Here, Papadakis et al. show that artificial optical magnetism can also be achieved with planar multilayer metamaterials.

## Introduction

In the optical spectral range, the magnetic response of most materials, given by the magnetic permeability *μ*, is generally weak. This is famously expressed by Landau et al.^1^: “there is no meaning in using the magnetic susceptibility from the optical frequencies onward, and in discussing such phenomena, we must put *μ* = 1”. By contrast, in the optical regime, materials possess a diverse range of dielectric properties, expressed through the dielectric permittivity $\epsilon$, which can be positive, negative, or zero.

The weak magnetic response in natural materials has motivated a search for structures and systems that may exhibit magnetic properties arising from metamaterial design. Specifically, engineered displacement currents and conduction currents can act as sources of artificial magnetism when metamaterials are illuminated with electromagnetic fields^2^. Nonetheless, until now, the realization of such magnetic metamaterials has required rather complex resonant geometries^2–4^, including arrays of paired thin metallic strips^5,6^, split-ring resonators^7–9^ and fishnet metamaterials^10^—structures that require sophisticated fabrication techniques at optical frequencies.

In contrast, the dielectric properties of metamaterials may be engineered even in simple planar configurations of layered media. Hence, heterostructures of alternating metallic and dielectric layers, termed hyperbolic metamaterials (HMMs), have been explored intensively the last decade^11–13^ due to their anisotropic dielectric response that is described by the dielectric permittivity tensor $\epsilon _{{\mathrm{eff}}}$ = diag{$\epsilon _{\mathrm{o}}$, $\epsilon _{\mathrm{o}}$, $\epsilon _{\mathrm{e}}$}, where $\epsilon _{\mathrm{o}}$ and $\epsilon _{\mathrm{e}}$ are the ordinary and extraordinary components of the tensor, with $\epsilon _{\mathrm{o}}\epsilon _{\mathrm{e}}$ < 0. Such a peculiar dielectric response manifests itself in the hyperbolic dispersion for transverse magnetic (TM) waves (i.e., **k** ⋅ **H** = 0 whereas **k** ⋅ **E ≠** 0). Interesting phenomena such as negative refraction^11,14–18^ without the need of a negative refractive index, hyper-lensing^19^, extreme enhancement in the density of optical states^13^, and interface-bound plasmonic modes^20–25^ have been reported.

**Fig. 1:**
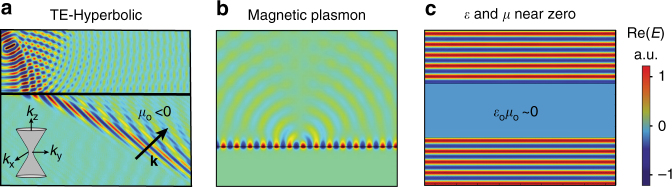
Implications of magnetic response in a planar geometry. **a** Transverse electric (TE) negative refraction of phase in a hyperbolic metamaterial with *μ*_o_ < 0 and *μ*_e_ > 0. The arrow indicates the direction of wavevector and the black line indicates the interface between air and the hyperbolic metamaterial. Inset: 3D isofrequency diagram for *μ*_o_ < 0, *μ*_e_ > 0. **b** TE magnetic plasmon at the interface between air and magnetic material (*μ* < 0), analogous to transverse magnetic polarized surface plasmon polaritons ($\epsilon$ < 0). **c** $\epsilon$ and *μ* near zero (EMNZ): a field propagating inside an EMNZ slab with vanishing phase advance

Nevertheless, all of the intriguing physics and applications for such layered HMMs have been limited to TM polarization, whereas phenomena related to transverse electric (TE) polarized waves (i.e., **k** ⋅ **E** = 0, whereas **k** ⋅ **H ≠** 0) have remained largely unexplored. Utilizing the effective magnetic response (i.e., $\mu _{{\mathrm{eff}}} = {\mathrm{diag}}\left\{ {\mu _{\mathrm{o}},\mu _{\mathrm{o}},\mu _{\mathrm{e}}} \right\} \neq {{\Bbb I}}$) is necessary to harness and control arbitrary light polarization (TE and TM). Namely, a multilayer system with $\epsilon _{\mathrm{o}}\epsilon _{\mathrm{e}}$ < 0 and *μ*_o_*μ*_e_ < 0 could allow polarization independent negative refraction (Fig. 1a) and excitation of TE surface waves (Fig. 1b), the magnetic counterpart of surface plasmon polaritons (SPPs). Furthermore, gaining control over the magnetic permeability in planar systems can yield impedance-matched epsilon-and-mu-near-zero (EMNZ) optical responses (Fig. 1c)^26^. Although it is straightforward to tailor the permittivity to cross zero in planar metamaterials^27^, a simultaneously EMNZ metamaterial at optical frequencies has not yet been demonstrated.

In previous reports, the effective magnetic permeability in planar layered media has been widely assumed to be unity^11–13,28^. We note that Xu et al.^18^ attributed their results of TM negative refraction and negative index to a negative magnetic parameter. This approach is valid in the case of isotropic media, however planar HMMs are extremely anisotropic, namely uniaxial. The index introduced in ref. ^18^ is the effective index of the mode excited in their experiment and is not directly associated with artificial magnetism. Furthermore, refraction switches to positive for TE polarization, similar to others reports^14–17^.

Here we propose a concept for tailoring the effective magnetic response within planar, unpatterned, one-dimensional (1D) multilayer structures. In contrast to previous generations of magnetic metamaterials with complex three-dimensional structures such as split-ring resonators^7–9^, fishnet structures^10^, and nanoparticles^29,30^, pattern-free multilayers are readily realizable with lithography-free thin-film deposition, greatly simplifying fabrication. We show theoretically and experimentally that the magnitude and sign of the permeability tensor may be engineered at will, enabling observation and use of TE polarization related phenomena in simple layered structures. We further identify implications that are associated with the observed artificial magnetism.

## Results

### Induced magnetic dipoles in planar systems

**Fig. 2:**
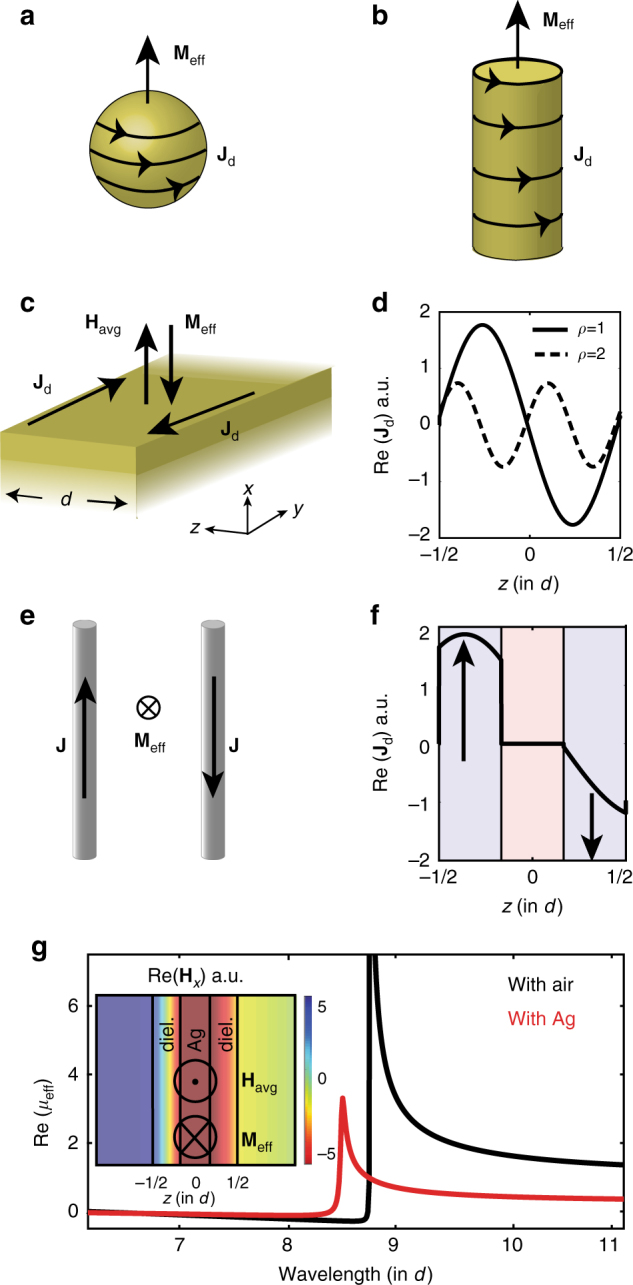
Concept of artificial magnetism in 3D, 2D, and 1D structures. A circulating current flow **J**_d_ induces a magnetization **M**_eff_ in all three cases: **a** dielectric nanoparticles (three-dimensional metamaterials), **b** dielectric nanorods (two-dimensional metamaterials), and **c** one-dimensional dielectric slabs. **H**_avg_ is the average magnetic field, which faces in the direction opposite to **M**_eff_. **d** Displacement current distribution at resonance for *ρ* = 1, *ρ* = 2, for a 90 nm slab of refractive index *n*_diel_ = 4.5. **e** Two infinite wires carrying opposite currents are equivalent to **f** two dielectric layers (blue shaded regions) separated by air (pink shaded region) in terms of their current distribution. Arrows in **f** indicate the direction of **J**_d_, which is anti-symmetric at resonance. **g** Effective permeability for two dielectric layers separated by air and silver. Inset: tangential magnetic field distribution at resonance: average magnetic field is opposite to **M**_eff_

A circulating electric current can create a magnetic dipole and is the key to inducing magnetism in magnet-free systems. Based on this principle, induction coils generate and induce magnetic flux, allowing to manipulate magnetic fields at radio frequencies (RFs). The same concept is widespread in metamaterials design^31,32^; similar to the RF regime, by properly shaping metamaterial elements to produce a circulating current flow, magnetic dipoles are induced. Dielectric nanoparticles^29,30,33–36^ and nanorods^37,38^ have been the building blocks for three (3D)- and two (2D)-dimensional magnetic metamaterial structures, respectively (Figs. 2a, b). We note that the magnetic response of these arrangements is sometimes incorporated into an equivalent, alternative, spatially dispersive permittivity. Although this is, in principle, always possible^1,39,40^, we stress that, similar to naturally occurring substances, described with a permittivity $\epsilon$ and a permeability *μ*, a metamaterial description based on ($\epsilon$, *μ*) allows for physical intuition and reduces complexity, especially when it is straightforward to relate the dielectric (magnetic) response with physical macroscopic electric (magnetic) moments. This can be particularly useful for uniaxial planar and unpatterned multilayers, studied in this paper.

We start by considering a single subwavelength dielectric slab of refractive index *n*_diel_ and thickness *d*. When illuminated at normal incidence (*z* direction in Fig. 2c), the displacement current ${\bf{J}}_{\mathrm{d}} = i\omega \epsilon _o\left( {n_{{\mathrm{diel}}}^{\mathrm{2}} - 1} \right){\bf{E}}$ induces a macroscopic effective magnetization ${\bf{M}}_{{\mathrm{eff}}} = 1{\mathrm{/}}2\mu _{o}{\int} \left( {{\bf{r}} \times {\bf{J}}_{\mathrm{d}}} \right) \cdot {\mathrm{d}{\it S}}$^1,37,41^. By averaging the magnetic field, ${\mathbf{H}}_{\rm avg} = {\int}_{ - d/2}^{d/2} {\mathbf{H}}(z){\mathrm{d}}{z}$, we use $\mu _{{\mathrm{eff}}} \simeq 1 + {\mathbf{M}}_{{\mathrm{eff}}}{\mathrm{/}}\left( {\mu _{o}}{\bf H}_{{\rm avg}} \right)$ to obtain an empirical closed-form expression for the magnetic permeability:

1 $$\mu _{\mathrm{eff}} \simeq 1 - \frac{n_{\mathrm{diel}}^{\mathrm{2}} - 1}{2n_{\mathrm{diel}}^{\mathrm{2}}}\left\{ { - 1 + \frac{{n_{\mathrm{diel}}\pi d{\mathrm{/}}\lambda }}{{{\mathrm{tan}}\left( {n_{{\mathrm{diel}}}\pi d{\mathrm{/}}\lambda } \right)}}} \right\}$$

By setting *n*_diel_ = 1, we recover the unity magnetic permeability of free space. From Eq. (1), we see that *μ*_eff_ diverges when tan(*n*_diel_*πd*/*λ*) = 0. This yields a magnetic resonant behaviour at free-space wavelengths *λ* = *n*_diel_*d*/*ρ*, with *ρ* = 1, 2, ... At these wavelengths, the displacement current distribution is anti-symmetric, as shown in Fig. 2d for *ρ* = 1, 2. This anti-symmetric current flow closes a loop in *y* = ±∞ and induces a magnetization **M**_eff_ that is opposite to the magnetic field of the incident wave (Fig. 2c), leading to a magnetic resonance. Eq. (1) enables estimating the design parameters for enhanced magnetic response; in the long-wavelength limit, only the fundamental and second order resonances, *λ* = *n*_diel_*d*, *n*_diel_*d*/2, play significant roles. In the visible and near-infrared regime, with layer thicknesses on the order of 10–100 nm, dielectric indices higher than *n*_diel_ ~ 2 are required for strong magnetic effects^42^. The same principle applies for grazing incidence, with the displacement current inducing a magnetic response in the extraordinary or, out-of-plane (*z*) direction. So far, we have shown that the circular shape designed to support a closed current loop is not a requirement for magnetic metamaterials. A planar structure suffices, for which the current loop closes in ± infinity.

In order to make this magnetic response significant, we extend this principle to multilayer configurations. We first examine the case of two infinite parallel wires in air, carrying opposite currents (Fig. 2e). Their net current distribution induces a magnetic moment that scales with their distance, as dictated by **M**_eff_ ∝ **r** × **J**. This is directly equivalent to a layered configuration composed of two high-index dielectrics separated by air. Their displacement current distribution can be anti-symmetric on resonance, as shown in Fig. 2f. By calculating their magnetic permeability *μ*_eff_, we confirm the magnetic character of this arrangement. As shown with the black curve in Fig. 2g, *μ*_eff_ strongly deviates from unity.

The planar geometry does not require that the two high-index layers be separated by air; any sequence of alternating high-low-high refractive index materials will induce the same effect. For example, replacing the air region with a layer of metal, with *n*_metal_ < 1 at visible wavelengths, does not drastically change the magnetic response. This is shown in Fig. 2g with the red curve for a separation layer of silver. Therefore, at optical frequencies, metals do not contribute significantly to the magnetic response in this planar configuration. This is in contrast to the gigahertz regime, where the conduction current in the metallic components of resonant structures has been necessary for strong magnetic effects^6–9^. From the magnetic field distribution shown in the inset of Fig. 2g, one can see that the average magnetic field faces in the direction opposite to the magnetization, expressing a negative magnetic response for the dielectric/silver unit cell (Supplementary Note 1).

### Combining hyperbolic dielectric and magnetic properties

**Fig. 3:**
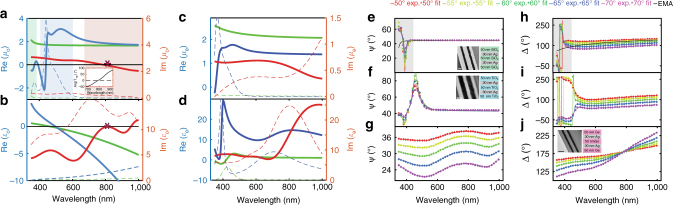
Experimental verification of non-unity magnetic permeability in dielectric/metal metamaterials. Experimentally determined **a** *μ*_o_, **b** $\epsilon _{\mathrm{o}}$, **c** *μ*_e_, **d** $\epsilon _{\mathrm{e}}$ for SiO_2_/Ag-green, TiO_2_/Ag-blue, Ge/Ag-red metamaterial. Shaded regions in **a** indicate the regime of magnetic resonances in *μ*_o_ for the studied metamaterials. Solid lines represent real parts while dashed lines represent imaginary parts. Asterisks in **a** and **b** indicate the $\epsilon _{\mathrm{o}}$ and *μ*_o_ near zero (EMNZ) wavelength for the Ge/Ag metamaterial. The EMNZ condition is confirmed by a vanishing phase of the transmission coefficient at the EMNZ wavelength, shown in the inset of **a**. **e**–**g** and **h**–**j** show the agreement between raw experimental data, Ψ and Δ respectively (which are the conventional ellipsometric angles), and the ellipsometric fitting, for the SiO_2_/Ag metamaterial in **e** and **h**, for the TiO_2_/Ag metamaterial in **f**, **i**, and for the Ge/Ag metamaterial in **g**, **j**. Shaded regions in **e**, **h** emphasize the disagreement between experimental data and the effective medium approximation (EMA). Insets in **e**, **f**, **j** show TEM images of the fabricated samples. The scale bar is 50 nm for **e**, **f** and 100 nm for **j**

Apart from the magnetic response described in the previous section, multilayer systems composed of metals and dielectrics have also been widely explored due to their distinct hyperbolic dielectric response for TM polarization. These systems are uniaxially anisotropic and, at wavelengths that are large compared with the unit cell, they exhibit an in-plane metallic response ($\epsilon _{\mathrm{o}}$ < 0) due to the metallic layers, whereas $\epsilon _{\mathrm{e}}$ > 0^11^. We show that it is possible to induce a significant additional magnetic response in planar dielectric/metal HMMs, if the dielectric layers are composed of high-index materials that are capable of supporting strong displacement currents at optical frequencies. Previously reported dielectric/metal HMMs have primarily featured dielectric layers with lower-refractive indices, such as LiF^43^, Al2O3^28,44,45^, and TiO2^13^. Figure 3 shows that, for layer thicknesses below ~ 50 nm, these lower-index dielectric/metal systems exhibit magnetic resonances in the ultraviolet (UV)-short wavelength visible regime.

Previous approaches used effective medium approximations (EMAs) for describing the dielectric response of dielectric/metal HMMs, such as the Maxwell Garnett theory^46^. However, such EMAs a priori assume a unity magnetic permeability along all coordinate directions. By contrast, a uniaxial system is most generally described in terms of both an effective permittivity tensor $\epsilon _{{\mathrm{eff}}}$ = diag{$\epsilon _{\mathrm{o}}$, $\epsilon _{\mathrm{o}}$, $\epsilon _{\mathrm{e}}$} and an effective permeability tensor *μ*_eff_ = diag{*μ*_o_, *μ*_o_, *μ*_e_}. In order to capture the magnetic dipole moments in multilayer structures, we use an exact parameter retrieval, which relaxes the $\mu _{{\mathrm{eff}}} = {\Bbb I}$ assumption^47^. We discuss and compare these different approaches in the Methods section.

### Experimental results

We fabricate multilayer structures by electron-beam evaporation and first measure the optical constants of the individual constituent layers with spectroscopic ellipsometry. We also determine their thicknesses with transmission electron microscopy (TEM). Hence, we are able to homogenize the layered metamaterials by assigning them effective parameters $\epsilon _{\mathrm{eff}}$ and $\mu _{\mathrm{eff}}$^47^, while taking into account fabrication imperfections. We then perform ellipsometric measurements of the full metamaterials and fit the experimental data with the effective parameters $\epsilon _{\mathrm{o}}$, $\epsilon _{\mathrm{e}}$, *μ*_o_, and *μ*_e_ in a uniaxial and Kramers–Kronig consistent model, whereas the total metamaterial thickness is held to the value determined through TEM. The fitting is over-determined as the number of incident angles exceeds the total number of fitted parameters (Supplementary Note 2).

The fabricated metamaterials are composed of SiO_2_/Ag, TiO_2_/Ag, and Ge/Ag alternating layers (TEM images and schematics in insets of Figs. 3e, f, j respectively). The indices of the selected dielectric materials at optical frequencies are $n_{{\mathrm{SiO}}_{\mathrm{2}}} \simeq 1.5$, $n_{{\mathrm{TiO}}_{\mathrm{2}}} \simeq 2$, and *n*_Ge_ ≃ 4–4.5. Figure 3a shows that increasing the dielectric index redshifts the magnetic resonance in the ordinary direction *μ*_o_; the SiO_2_/Ag metamaterial supports a magnetic resonance in the long-wavelength UV regime (~ 300 nm), whereas the TiO_2_/Ag and Ge/Ag metamaterials exhibit resonances in the blue (450 nm) and red (800 nm) part of the spectrum, respectively. The enhanced absorption in Ge at optical frequencies leads to considerable broadening of the Ge/Ag metamaterial magnetic resonance, yielding a broadband negative magnetic permeability for wavelengths above 800 nm. As expected, the losses in *μ*_o_ are increased at the magnetic resonance frequency for all investigated heterostructures, similar to previous reports on artificial magnetism with split-ring resonators and other magnetic metamaterials^48–50^.

The presence of Ag induces a negative ordinary permittivity $\epsilon _{\mathrm{o}}$ (Fig. 3b), which, for the Ge/Ag metamaterial, becomes positive above 800 nm due to the high-index of Ge. Notably, $\epsilon _{\mathrm{o}}$ crosses zero at 800 nm, similar to *μ*_o_, as emphasized with the asterisks in Figs. 3a, b. Thus, the Ge/Ag metamaterial exhibits an EMNZ response at optical frequencies. The EMNZ condition is confirmed by transfer-matrix analytical calculations of the physical multilayer structure. As shown in the inset of Fig. 3a, the phase of the transmission coefficient vanishes at the EMNZ wavelength, demonstrating that electromagnetic fields propagate inside the metamaterial without phase advance^26^.

By comparing *μ*_*o*_ and *μ*_*e*_ in Figs. 3a, c, respectively, one can infer that increasing the dielectric index leads to enhanced magnetic anisotropy. The parameter *μ*_e_ only slightly deviates from *μ*_o_ for the SiO_2_/Ag metamaterial, while the deviation is larger for the TiO_2/_Ag one. For the Ge/Ag metamaterial, *μ*_e_ remains positive beyond 800 nm, while *μ*_o_ < 0, indicating magnetic hyperbolic response for TE polarization. Furthermore, all three heterostructures exhibit hyperbolic response for TM polarization, with $\epsilon _{\mathrm{o}}$ < 0 and $\epsilon _{\mathrm{e}}$ > 0 (Figs. 3b, d). Consequently, the Ge/Ag metamaterial possesses double hyperbolic dispersion.

Figures 3e–j demonstrate the excellent agreement between fitting and raw experimental data, where the parameters Ψ and Δ correspond to the conventional ellipsometric angles (Methods). In Figs. 3e, h, we also provide a Maxwell Garnett EMA-based fit for the SiO_2_/Ag metamaterial. The EMA fails to reproduce the experimentally measured features, in both Ψ and Δ (gray-shaded regions in Figs. 3e, h), which correspond to magnetic permeability resonances. Similar EMA-based fits for the TiO2/Ag and Ge/Ag metamaterials lead to large disagreement with the experimental data across the whole visible-near-infrared spectrum and are, thus, omitted. This disagreement is expected, as the EMA approach is based on the assumption that the electric field exhibits negligible or no variation within the lattice period^46^, which does not apply to high-index dielectric layers.

It should be noted that the dielectric hyperbolic response $\epsilon _{\mathrm{o}}\epsilon _{\mathrm{e}}$ < 0 is broadband in planar systems, as seen in Figs. 3b, d. In contrast, the magnetic permeabilities deviate from unity in a resonant manner along both coordinate directions *μ*_o_ and *μ*_e_, thereby making TE polarization-based phenomena more narrow band in nature.

### Beyond *μ*_eff_ ≠ 1 and TE polarization effects

**Fig. 4:**
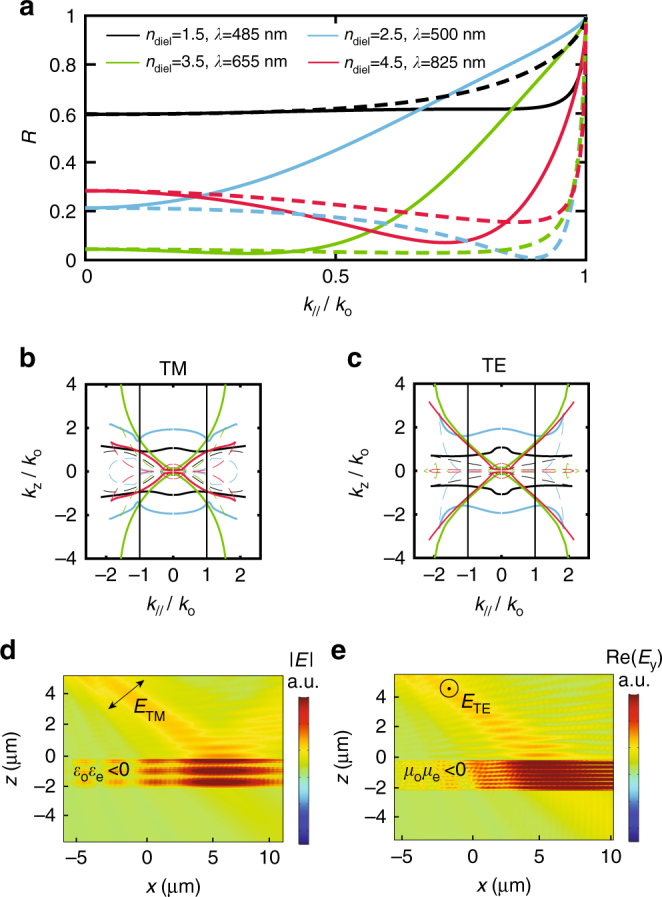
Bulk propagating modes in magnetic hyperbolic layered metamaterials. Analytical calculations of **a** reflectance and **b**, **c** isofrequency diagrams for a metamaterial consisting of five alternating layers of dielectric *n*_diel_: 55 nm/Ag: 25 nm. Solid lines in **a** correspond to TE polarization whereas dashed lines correspond to TM polarization. Solid lines in **b**, **c** correspond to real parts, whereas dashed lines correspond to imaginary parts. Vertical black lines in **b**, **c** indicate the maximum free space in-plane wavenumber *k*_//_ = *k*_o_. Color code is the same for **a**–**c**. **d**, **e** Numerical simulation of a fifty-five layers dielectric (*n*_diel_ = 4)/Ag multilayer metamaterial. The surrounding medium has index *n*_sur_ = 1.55, allowing coupling of high-*k* modes. We increased the number of layers for clear visibility of field localization inside the structure. Strong field localization is the consequence of **d** dielectric hyperbolic dispersion for TM polarization ($\epsilon _{\mathrm{o}}\epsilon _{\mathrm{e}}$ < 0) and **e** magnetic hyperbolic dispersion for TE polarization (*μ*_o_*μ*_e_ < 0)

**Fig. 5:**
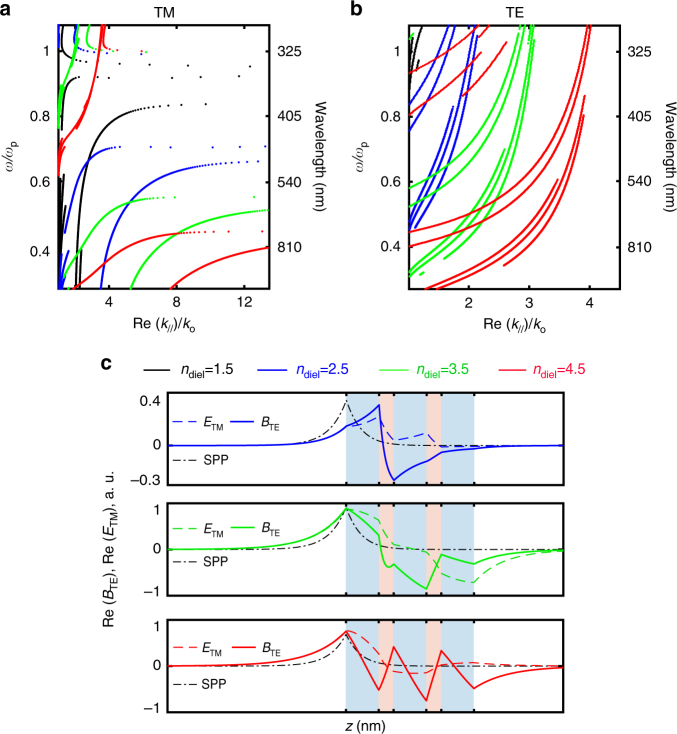
Surface waves in magnetic hyperbolic layered metamaterials. **a** TM and **b** TE surface wave dispersion for a metamaterial consisting of five alternating layers of dielectric *n*_diel_: 55 nm/Ag: 25 nm. **c** Field profiles (incidence from the left) and comparison with a surface plasmonic polariton (SPP) on an equivalent Ag slab (black dotted line). Calculations in **c** correspond to a wavelength of 620 nm for *n*_diel_ = 2.5, 880 nm for *n*_diel_ = 3.5 and 1100 nm for *n*_diel_ = 4.5. Blue shaded regions in **c** indicate dielectric layers whereas pink shaded regions indicate Ag

In the previous sections we established, theoretically and experimentally, that dielectric/metal layered systems may be described with an effective magnetic permeability that deviates from unity across all coordinate directions. The purpose of introducing this parameter is to build a simple and intuitive description for understanding and predicting new phenomena, such as TE polarization response in planar systems. In what follows we discuss how the non-unity and, in particular the negative and anisotropic magnetic response that we demonstrated (Fig. 3) manifests itself in the characteristics of TE-polarized propagating modes (Fig. 4) and surface waves (Fig. 5).

We use an example system of dielectric/silver alternating layers, similar to the one we investigate experimentally. To emphasize that enhanced magnetic response at optical frequencies requires high-index dielectrics, we let the refractive index of the dielectric material *n*_diel_ vary. The calculations and full-wave simulations presented here are performed in the actual, physical, multilayer geometry (Figs. 4a, d, e and 5) and compared with the homogeneous effective slab picture ($\epsilon _{{\mathrm{eff}}}$, *μ*_eff_—Figs. 4b, c). This helps assess the validity of our model and emphasize the physicality of the magnetic resonances.

First, we perform transfer-matrix calculations for the example multilayer metamaterial and we show in Fig. 4a the angle dependence for TE and TM reflectance. The strong angle dependence for TM polarization is well understood in the context of an equivalent homogeneous material with anisotropic effective dielectric response $\epsilon _{\mathrm{o}}\epsilon _{\mathrm{e}}$ < 0. Bulk TM modes experience dispersion

2 $$\frac{{k_x^2 + k_y^2}}{{\epsilon _{\mathrm{e}}(\omega ,{\bf{k}})\mu _{\mathrm{o}}(\omega ,{\bf{k}})}} + \frac{{k_z^2}}{{\epsilon _{\mathrm{o}}(\omega ,{\bf{k}})\mu _{\mathrm{o}}(\omega ,{\bf{k}})}} = k_{\mathrm{o}}^2$$

where *k*_o_ = *ω*/*c*. This dispersion is hyperbolic, as shown with isofrequency diagrams in Fig. 4b. Losses and spatial dispersion perturb the perfect hyperbolic shape^12^. In contrast to the TM modes, TE bulk modes interact with the magnetic anisotropy through the dispersion equation

3 $$\frac{{k_x^2 + k_y^2}}{{\epsilon _{\mathrm{o}}(\omega ,{\bf{k}})\mu _{\mathrm{e}}(\omega ,{\bf{k}})}} + \frac{{k_z^2}}{{\epsilon _{\mathrm{o}}(\omega ,{\bf{k}})\mu _{\mathrm{o}}(\omega ,{\bf{k}})}} = k_{\mathrm{o}}^2$$

which is plotted in Fig. 4c. For small wavenumbers (*k*_//_/*k*_o_ < 1) and small dielectric indices *n*_diel_, the isofrequency diagrams are circular, in other words, isotropic. This agrees well with our experimental results; as shown in Figs. 3a, c, for the SiO_2_/Ag metamaterial, ordinary and extraordinary permeabilities do not drastically deviate from each other. Increasing the dielectric index opens the isofrequency contours, due to enhanced magnetic response in the ordinary direction (*μ*_o_), which leads to magnetic anisotropy. We note that the displayed wavelengths are selected at resonances of *μ*_o_. Open TE polarization isofrequency contours for *n*_diel_ ≥ 2 are also consistent with experimental results; as shown in Fig. 3 for TiO_2_ and Ge-based metamaterials, increasing *n*_diel_ enhances the anisotropy. This also agrees well with the picture of the physical multilayer structure, as shown in Fig. 4a; the TE reflectance indeed exhibits extreme angle dependence for increasing dielectric index. Strikingly, we observe a Brewster angle effect for TE polarization, which is unattainable in natural materials due to unity magnetic permeability at optical frequencies^51^.

An open isofrequency surface can yield an enhancement in the density of optical states relative to free space. Physically, this may lead to strong interaction between incident light and a hyperbolic structure, and enhanced absorption when it is possible to couple to large wavenumbers from the surrounding medium^52,53^. So far, only TM polarization has been considered to experience this exotic hyperbolic response in planar dielectric/metal metamaterials, due to $\epsilon _{\mathrm{o}}\epsilon _{\mathrm{e}}$ < 0^12,13,28^. Based on the open isofrequency surfaces for both TE and TM polarizations in Figs. 4b, c, a high-index dielectric/metal multilayer metamaterial may exhibit distinct frequency regimes of double, that is, simultaneously TE and TM polarization, hyperbolic-like response. To confirm this, we perform finite element simulations of a (*n*_diel_ = 4)/silver multilayer metamaterial for both linear polarizations and set the index of the surrounding medium to *n*_sur_ = 1.55 to allow coupling to larger wavenumbers. To facilitate visualizing the interaction between the fields and the metamaterial, we consider a thick structure consisting of fifty-five layers. Without loss of generality, we carry out the simulation in the low loss limit to unveil the physics while avoiding side effects due to losses. Figure 4d demonstrates the well-known TM hyperbolic response since the electric field is strongly localized within the multilayer. Switching the polarization to TE (Fig. 4e), we observe similar hyperbolic behaviour, which, however, cannot be attributed to dielectric anisotropic response as the electric field only experiences the in-plane dielectric permittivity $\epsilon _{\mathrm{o}}$ (Eq. (3)). The TE enhanced absorption is associated with the *μ*_o_*μ*_e_ < 0 condition^54^; the number of TE modes supported by this metamaterial in this frequency regime is drastically increased (Supplementary Note 3).

Finally, we investigate surface wave propagation in our example system of a layered dielectric (*n*_diel_)/silver metamaterial. We do so by utilizing the transfer matrix mode condition *m*_11_ = 0^55^, which we implement numerically using the reflection pole method^56^. In order to ensure interface-localized propagation with fields decaying in air and in the metamaterial, we impose an additional constraint for the waves to be located in the optical band gaps of both bounding media (Supplementary Note 4).

Figure 5a displays the dispersion for TM polarization. The identified surface waves bear similarity to typical SPPs on metallic interfaces^22,57^ and to plasmonic waves in dielectric/metal waveguides and systems^24^. Their plasmonic nature is evident as their dispersion asymptotically approaches the surface plasma frequency, similar to SPPs. We show in Fig. 5c their field distribution (dashed lines), and compare to SPPs on an equivalent silver slab (black dotted lines). Such TM surface waves on metamaterial interfaces are often associated with an effective negative dielectric response^20,21,25^. This is consistent with our effective dielectric and magnetic model; as we showed experimentally in Fig. 3b, the ordinary permittivity is negative $\epsilon _{\mathrm{o}}$ < 0.

Performing the same analysis for TE polarized waves, we find that TE surface-bound modes also exist (Fig. 5b). Their dispersion is parabolic, resembling that of Tamm states in photonic crystals^25,58^. However, here we show that they also exist in the subwavelength metamaterial limit and can coexist with typical TM plasmonic surface waves. TE polarized Tamm states have been previously associated only qualitatively with some arbitrary negative net magnetic response^25^. Here we confirm this hypothesis and explicitly connect the dispersion of Tamm states in planar metamaterials to values of magnetic permeabilities that were experimentally measured (Fig. 3). We further identify their physical origin, which is the strong displacement current supported in high-index dielectric layers with a loop-like distribution on resonance. These TE surface waves emerge in the visible regime for dielectric layers with refractive index *n*_diel_ ≥ 2 (Fig. 5b), at frequencies where the metamaterial exhibits a negative effective magnetic response. For this reason, these states may be seen as magnetic plasmons.

The frequency regimes in which double surface waves are supported demonstrate the possibility of exciting TM polarized plasmonic modes simultaneously with their TE counterparts in dielectric/metal pattern-free multilayers.

## Discussion

In conclusion, we have shown that non-unity effective magnetic permeability at optical frequencies can be obtained in 1D-layered systems, arising from displacement currents in dielectric layers. This makes it possible to tailor the magnetic response of planar HMMs, which have been previously explored only for their dielectric permittivity features. We experimentally demonstrated negative in-plane magnetic permeability in planar structures, which can lead to double HMMs. By studying bulk and surface wave propagation, we have identified frequency regimes of a rather polarization-insensitive response. We reported the existence of TE polarized magnetic surface plasmons, attributed to the negative magnetic permeability, which are complementary to typical TM polarized surface plasmonic modes in materials with negative dielectric permittivity. The results reported here can open new directions for tailoring wave propagation in artificial magnetic media in significantly simplified layered systems. We anticipate the reported findings to enable the generalization of the unique properties of plasmonics and HMMs, previously explored for TM polarized waves and negative permittivity media, for unpolarized light at optical frequencies.

## Methods

### Relaxing the *μ*_eff_ = 1 constraint

Here we briefly discuss how our computational method allows relaxing the previously made *μ*_eff_ = 1 assumption. The most extensively used approach for describing the effective response of hyperbolic multilayer metamaterials is the Maxwell Garnett EMA^11^ (and references therein)^12,13^. Based on this approach, the in-plane dielectric permittivity is given by $\epsilon _{\mathrm{o}}$_,MG_ = *f*$\epsilon _{\mathrm{m}}$ + (1 − *f*)$\epsilon _{\mathrm{d}}$ and the out-of-plane extraordinary permittivity is $\epsilon _{{\mathrm{e,MG}}}^{ - 1} = f\epsilon _{\mathrm{m}}^{ - 1} + (1 - f)\epsilon _{\mathrm{d}}^{ - 1}$, where *f* is the metallic filling fraction^46^, while *μ*_eff_ is a priori set to unity. Another commonly used approach is the Bloch formalism, based on which, a periodic A-B-A-B… superlattice is described with a Bloch wavenumber^55^. This wavenumber is directly translated to an effective dielectric permittivity^59^, while also assuming *μ*_eff_ = 1. These approaches are useful and simple to use, however, they are both based on the assumption of an infinite and purely periodic medium, ignoring the finite thickness of realistic stacks.

By contrast, metamaterials other than planar ones, which are, in general, more structurally complex, such as split-ring resonators^7–9,60^, nanoparticles^30^, and fishnet structures^61,62^, are modeled with exact S-parameter retrieval approaches^63,64^. S-parameter retrievals solve the inverse problem of determining the effective dielectric permittivity and magnetic permeability, $\epsilon _{{\mathrm{eff}}}$ and *μ*_eff_, respectively, of a homogeneous slab with the same scattering properties, namely transmission *T* and reflection *R* coefficients, as the arbitrary inhomogeneous, composite metamaterial system of finite thickness *d*.

**Fig. 6:**
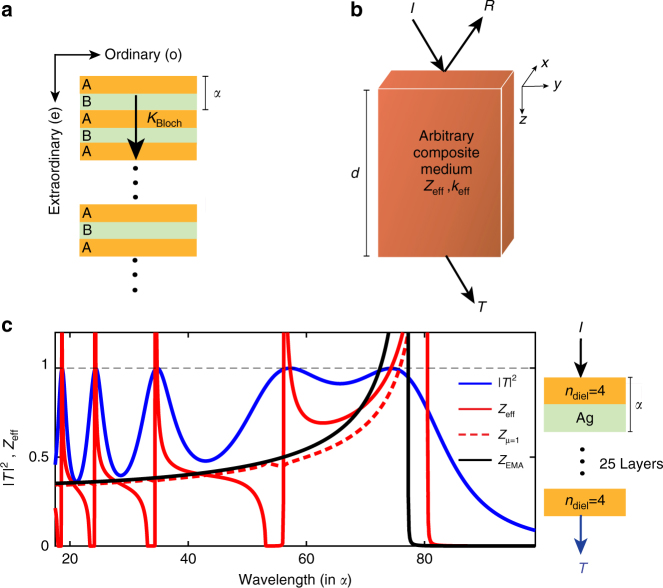
Comparison between effective medium theory (EMA) and Bloch approaches and S-parameter retrieval. **a** EMA and Bloch formalisms (for infinite periodic arrangements), *α* is the lattice period and *K*_Bloch_ is the Bloch wavenumber, **b** general concept of S-parameter retrievals that take finite total thickness *d* into account. *T* and *R* are the transmitted and reflected amplitudes and *Z*_eff_ and *k*_eff_ are the effective impedance and wavenumber. **c** Impedance-matching sanity check at normal incidence for a twenty-five layers dielectric/metal metamaterial, for *n*_diel_ = 4. The transmittance $\left| T \right|^2$ calculation (blue solid line) was performed with the transfer-matrix formalism^55^ for the physical multilayer system in the lossless limit. The dielectric and magnetic effective model $\left( {Z_{{\mathrm{eff}}} = \sqrt {\mu _{\mathrm{o}}{\mathrm{/}}\epsilon _{\mathrm{o}}} } \right)$ (red solid line) accurately captures the resonances unlike the non-magnetic approach $\left( {Z_{\mu _{{\mathrm{eff}}}{\mathrm{ = 1}}}} \right)$ (red dashed line) and the Maxwell Garnett EMA (black solid line)

By lifting the constraint of an infinite medium, both transmission *T* and reflection *R* coefficients can be computed and used in S-parameter approaches. This allows obtaining an effective wavenumber *k*_eff_ together with an effective impedance *Z*_eff_^63,64^. These parameters are then used to decouple the effective permittivity from the permeability through $k_{{\mathrm{eff}}} = \sqrt {\epsilon _{{\mathrm{eff}}}\mu _{{\mathrm{eff}}}} \frac{\omega }{c}$ and $Z_{{\mathrm{eff}}} = \sqrt {\frac{{\mu _{{\mathrm{eff}}}}}{{\epsilon _{{\mathrm{eff}}}}}}$. By contrast, Bloch-based approaches^55,59^ only consider a Bloch wavenumber *K*_Bloch_ (based on periodicity), with no other information available for allowing decoupling *μ*_eff_ from $\epsilon _{{\mathrm{eff}}}$. Both the Maxwell Garnett result^46^ and its Bloch-based generalizations (for example^59^) are based on the assumption that *μ*_eff_ = 1. A schematic comparison between the two approaches is shown in Figs. 6a, b.

Contrary to the extensive use of EMAs, we use the S-parameter approach to describe dielectric/metal multilayer metamaterials of finite thickness. By letting the magnetic permeability *μ*_eff_ be a free parameter, instead of a priori setting *μ*_eff_ = 1, we obtain magnetic resonances at wavelengths where magnetic dipole moments occur, as demonstrated in Figs. 2f, g. This confirms the physicality of the non-unity *μ*; magnetic resonances arise at wavelengths where the system supports loop-like current distributions.

By accounting for the uniaxial anisotropy in planar heterostructures, we obtain both the ordinary and the extraordinary permeabilities *μ*_o_ and *μ*_e_, together with their dielectric permittivity counterparts, $\epsilon _{\mathrm{o}}$ and $\epsilon _{\mathrm{e}}$. As a sanity check, we first consider homogeneous metallic and dielectric slabs with known dielectric permittivity $\epsilon _{\mathrm{o}}$ = $\epsilon _{\mathrm{e}}$ and *μ*_o_ = *μ*_e_ = 1, which we recover upon application of our retrieval^47^.

Another way to establish the validity of the effective parameters is to perform an impedance-matching sanity check in the low loss limit. Based on electromagnetic theory, the impedance of a structure at normal incidence, $Z_{{\mathrm{eff}}} = \sqrt {\frac{{\mu _{\mathrm{o}}}}{{\epsilon _{\mathrm{o}}}}}$, must be unity at transmittance $\left| T \right|^2$ maxima. As seen in Fig. 6c, the retrieved parameters $\epsilon _{\mathrm{o}}$ and *μ*_o_ accurately describe the scattering properties of planar dielectric/metal arrangements of finite thickness. By contrast, not accounting for a magnetic permeability leads to inaccurate prediction of transmittance maxima. This is seen both by our S-retrieval-based approach while setting a priori the magnetic permeability to unity (*Z*_*μ*=1_), and with the traditional EMA; both approaches fail to predict the resonances (Supplementary Note 5).

By sweeping the angle of incidence from 0 to 90 degrees, i.e., by varying the in-plane wavenumbers *k*_//_, we obtain angle-independent, local material parameters for the systems we consider^47^. This makes ellipsometry a suitable method to experimentally characterize our metamaterials in terms of local material tensorial parameters *μ*_eff_ and $\epsilon _{{\rm{eff}}}$. For larger $k_{//} \gg \frac{\omega }{c}$, dielectric/metal arrangements exhibit some degree of spatial dispersion, due to the plasmonic nature of the metallic layers^65^. This effect is distinct from the magnetic resonances we investigate, which are the result of induced magnetic dipole moments^2^. Spatial dispersion is fully accounted for in the results presented here. This is done by extending our previous approach^47^ to consider as a free parameter not only the magnetic permeability, but also spatial dispersion in the form of wavenumber (*k*_//_) dependence (see discussion pertaining to Figs. 4b, c). Furthermore, as seen by the experimentally confirmed effective parameters discussed in Fig. 3, all constituent permittivity and permeability components ($\epsilon _{\mathrm{o}}$, $\epsilon _{\mathrm{e}}$, *μ*_o_, *μ*_e_) are passive and causal and have positive imaginary parts with no antiresonance artifacts. Such artifacts are often associated with weak form of spatial dispersion (see^66^ and discussion in^67^ and^68^ among others).

Other approaches are also able to capture this artificial magnetic response by accounting for an effective permeability in multilayer metamaterials. The general field averaging scheme introduced by Smith and Pendry^41^ captures the magnetic permeability we introduce, as discussed with regards to Fig. 2 and Eq. (1). This scheme has been implemented in the work by Watanabe et al.^51^. Another method can be found in^69^ and in references therein. Both of these approaches, however, must be used with caution as they do not explicitly account for spatial dispersion; in contrast, spatial dispersion is taken into account by being considered as a free parameter in Mota et al.^65^ and Papadakis et al.^47^.

### Sample preparation

We prepared the layered SiO_2_ metamaterials by electron-beam evaporation onto Ge substrates. The Ge/Ag sample was deposited on a Si substrate to avoid interface effects with the first Ge layer. All samples discussed in this work contain layers of 30 nm of Ag. Each Ag layer was deposited by first seeding a 2 nm AgO layer that was reduced to Ag under vacuum for obtaining smoother interfaces^70^. Atomic force microscopy measurements indicated Ag roughness of 2.13 nm. The thickness of the Ge, TiO_2_ and SiO_2_ was aimed to be 40 nm. Thickness deviations were measured with TEM, varying ±20 nm. TEM images are displayed in Figs. 3e, f, j.

### Ellipsometry

The ellipsometrically measured parameters Ψ and Δ correspond to the relative change of polarization state in amplitude and phase, respectively, of a reflected beam off a sample. With respect to the complex reflection coefficients for TM and TE polarization, $\tilde R_{{\mathrm{TM}}}$ and $\tilde R_{{\mathrm{TE}}}$, Ψ and Δ are defined as $\tilde R_{{\mathrm{TM}}}{\mathrm{/}}\tilde R_{{\mathrm{TE}}} = {\mathrm{tan}}\left( {{\Psi}} \right)e^{i{\mathrm{\Delta }}}$.

### Data availability

All relevant data are available from the authors upon request.

## Electronic supplementary material

Supplementary Information
