# Supplementary material for: Optical magnetism in planar metamaterial heterostructures
Source: Nat Commun. 2018 Jan 18;9:296. doi: 10.1038/s41467-017-02589-8 (PMC5773539; doi:10.1038/s41467-017-02589-8)
Supplement: Supplementary file 1 — Supplementary Information [file 41467_2017_2589_MOESM1_ESM.pdf]

# Supplementary Information

## Supplementary Note 1: Field profiles on and off resonance

The effective magnetic permeability discussed in Figs. 2, 3 of the main text originates from loop-like displacement current in dielectric regions. Let us examine the dielectric ($n_{\text{diel}} = 4.5$) /Ag/dielectric structure discussed in Fig. 2g of the main text. At the magnetic permeability resonance, the displacement current exhibits an anti-symmetric distribution, as shown in the Supplementary Figure 1a (similarly to Fig. 2f of the main text, which pertains to the ($n_{\text{diel}} = 4.5$) /air/dielectric case). The circular current loop leads to a non-zero effective magnetization given by: $\mathbf{M_{eff}} = \frac{1}{2}\mu_{\mathbf{o}} \int \mathbf{r} \times \mathbf{J_d} \cdot dS$ [1-3]. We also calculate the external tangential magnetic field profile at resonance, which changes sign at the air/metamaterial interface and becomes positive ($+x$) inside the metamaterial (Supplementary Figure 1b). This leads to an average magnetic field $\mathbf{H_{avg}}$, which faces in the opposite direction ($+x$) to the effective magnetization $\mathbf{M_{eff}}$ ($-x$). This shows the effective diamagnetic character in the negative side of the magnetic resonance [4] (See Fig. 2g of the main text), based on the definition of the magnetic permeability: $\mu_{\text{eff}} = 1 + M_{\text{eff}} / (\mu_{\text{eff}} H_{avg})$. Furthermore, the magnetic field null at the air/metamaterial interface is a characteristic of a perfect magnetic conductor (PMC), exhibiting a resonating magnetic permeability, which agrees with the picture of an effective equivalent slab with dielectric and magnetic properties, as the computed $\mu_{\text{eff}}$ exhibits a resonance (Fig. 2g of the main text). Contrary to the on resonance case, the displacement current is no longer anti-symmetric off resonance (Supplementary Figure 1c-please note the colour bar). Deviation of the displacement current distribution from the well defined anti-symmetric loop shape leads to an effective magnetization that does not considerably deviate from unity. This is evident in Supplementary Figure 1d; the incident magnetic field only weakly interacts with the metamaterial, as its value is almost unchanged with respect to the illumination side (left).

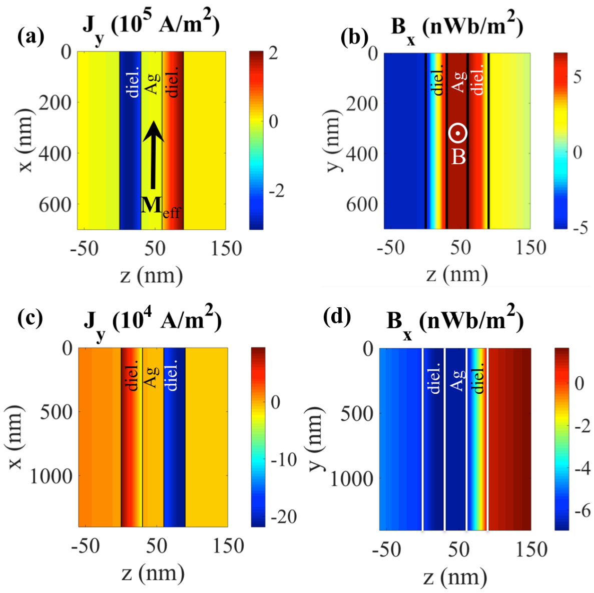


Supplementary Figure 1: **Displacement currents and magnetic field profiles.** Displacement current distribution for a ($n_{diel}$=4.5): 30 nm/Ag: 30 nm/($n_{diel}$=4.5): 30 nm multilayer metamaterial. Panel (a) corresponds to the on resonance case whereas panel (c) corresponds to an off resonance frequency, hence, the displacement current is not antisymmetric in (c). Panels (b), (d) show the tangential magnetic field profile on and off resonance respectively: the average external magnetic field in (b) is opposite to the effective magnetization $\mathbf{M}_{eff}$ on-resonance, indicating $\mu_{eff}$<0. Results pertain to normal incidence and illmination from the left.

**Supplementary Note 2:** Experimental characterization with spectroscopic ellipsometry and sample preparation details

Complementary to Figs. 3e, f, j of the main text, we provide here enlarged versions of the transmission electron microscopy (TEM) images of the samples under consideration.

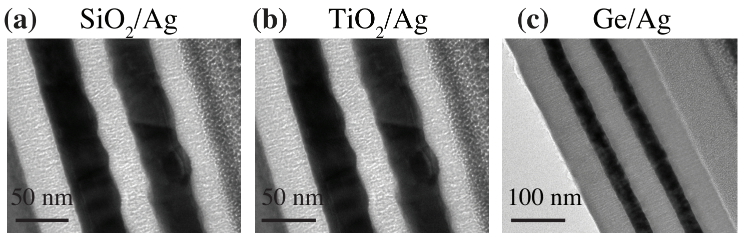


Supplementary Figure 2: **Transmission electron microscopy (TEM) images of fabricated samples.** (a) $SiO_2$/Ag metamaterial, (b) $TiO_2$/Ag metamaterial, (c) Ge/Ag metamaterial.

We characterize the optical properties of each constituent layer via spectroscopic ellipsometry on reference samples, and determine the exact thickness of each layer from the TEM images. We are, thus, able to homogenize our metamaterials while taking into account fabrication and materials imperfections. We perform two types of homogenizations, which we then use as models for fitting the ellipsometric data on the full metamaterials: the first one pertains to our parameter retrieval approach that takes into account the magnetic response of planar metamaterials [5] (calculating $\varepsilon_o, \varepsilon_e, \mu_o, \mu_e$). We refer to this model as the "effective slab" model in Supplementary Fig. 3, as it accounts for the finite thickness of our metamaterials. By contrast, the second fitting uses effective medium approximation (EMA), which

pertains to an infinite arrangement. Particularly, we use the Maxwell Garnett result EMA result [6].

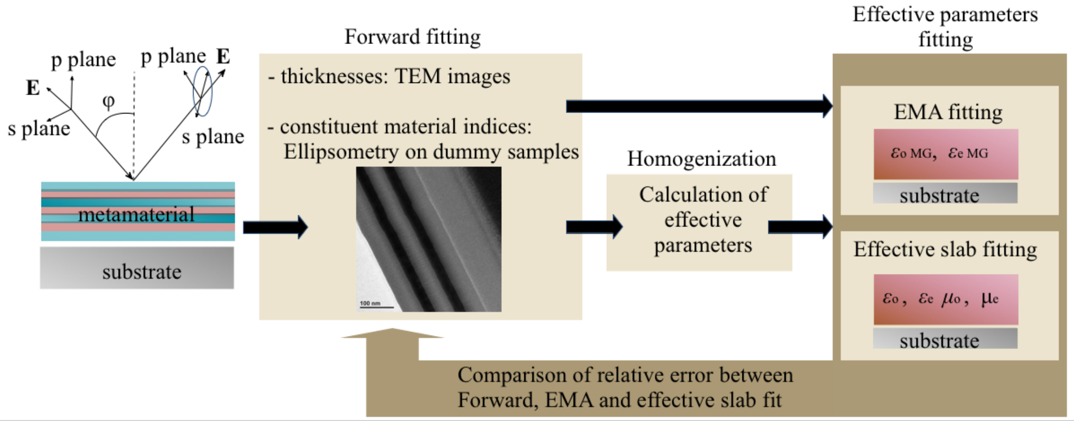


Supplementary Figure 3: **Experimental approach with spectroscopic ellipsometry**. Measurements are taken on the full metamaterial stacks. The forward fitting pertains fitting the experimental data using the physical multilayer structure. We perform calculations of effective parameters ($\varepsilon_{o}, \varepsilon_{e}, \mu_{o}, \mu_{e}$ and $\varepsilon_{oEMA}, \varepsilon_{eEMA}$) for the metamaterials while accounting for experimentally measured thicknesses through transmission electron microscopy (TEM) and experimentally determined optical constants of the constituent materials through spectroscopic ellipsometry on reference samples. We then perform fitting of the experimental data on the full metamaterials using the effective medium theory (EMA) model and the effective slab model that accounts for magnetic effects.

We then take spectroscopic ellipsometry measurements on the full metamaterials, for angles of incidence 50° to 70° with a step of 5°. By taking measurements for five angles of incidence, and fitting four effective parameters: $\varepsilon_{o}, \varepsilon_{e}, \mu_{o}, \mu_{e}$ in the effective slab model, the fitting problem is always over-determined. The same holds for the fitting performed using the EMA approach ($\varepsilon_{oEMA}, \varepsilon_{eEMA}$). The ellipsometric fitting for all the samples discussed in this work is performed in the commercially available VASE system (J. A. Woollam Co.), and the effective parameters are all modeled with generalized Drude, Lorenz and Gaussian oscillators to ensure Kramers-Kronig consistency. The VASE system utilizes a Levenberg-Marquardt regression algorithm for the fit. We use the META6 model of WVASE to incorporate the uniaxial anisotropy.

**Supplementary Note 3:** Reflection pole method for detecting eigenmodes and counting states

Here we explain the reflection pole method [7], based on which we detect the eigenmodes supported in the metamaterials we study, relevant to Figs. 4, 5 of the main text. Using the transfer matrix formalism for layered media [8], an eigenmode is an excitation ($\omega, k_{//}$) that is a pole of the complex transmission coefficient given by $t(\omega, k_{//}) = 1/m_{11}(\omega, k_{//})$, where $m_{11}$ is the 1-1 tensor element of the transfer matrix [8]. Based on the residue theorem of complex analysis, at the zeros of a complex function $m_{11}(k_{//})$, it's phase shifts by $\pi$. We take the derivative $\partial m_{11}(k_{//}/k_o) \big/ \partial(k_{//}/k_o)$, which, as shown in Supplementary Fig. 4, resonates at the zero of $m_{11}$. The real part of the effective index of the eigenmode is given by the position of the resonance, while its imaginary part is given by the half-width-half-maximum (FWHM). For more details see [7] or [9].

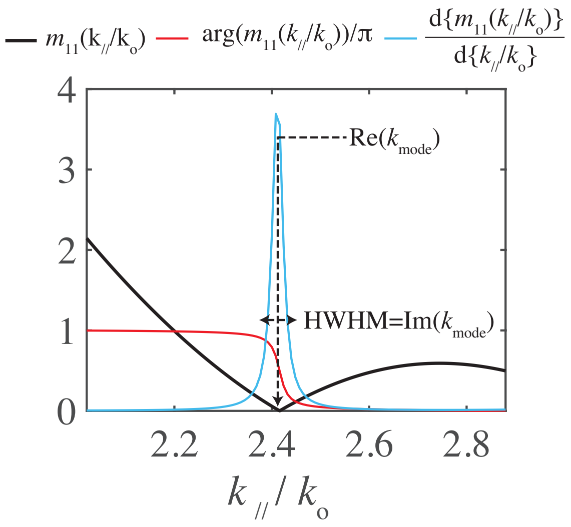


Supplementary Figure 4: **Reflection pole method.** An optical mode is detected using the reflection pole method. The transfer matrix element $m_{11}(k_{//})$ vanishes, hence its phase $\arg(m_{11}(k_{//}))$ shifts by π. By detecting the peaks of the derivative of $m_{11}(k_{//})$ with respect to $k_{//}$ we obtain both real and imaginary parts of the effective index of the optical mode. The real part corresponds to the resonance position whereas the imaginary part corresponds to the half-width-half-maximum (HWHM) of the resonance.

We consider in-plane normalized wavenumbers $k_{//}/k_o \in [0, n_{sur}]$, for radiation (bulk) modes, while $k_{//}/k_o > n_{sur}$ for bounded (surface) modes, where by $n_{sur}$ we refer to the refractive index of the surrounding medium. In order to explicitly show the increase in the number of TE-polarized electromagnetic states supported by metamaterials that exhibit magnetic resonances at visible wavelengths, like the one discussed in Fig. 4e of the main text, we start with the analytical equation for the density of states, pertaining to planar structures:

$$\rho(\omega,z,\vec{k}) = \frac{3}{2|p|^2 k_1^3}\frac{k_x}{k_z}\left\{\frac{1}{2}p_{par}{}^2(1-r^p e^{2ik_z z})k_z{}^2 + p_{norm}{}^2(1+r^p e^{2ik_z z})k_x{}^2 + \frac{1}{2}p_{par}{}^2(1+r^s e^{2ik_z z})k_1{}^2\right\} \quad (1)$$

where $r^s$ and $r^p$ refer to the complex reflection coefficients for TE (s) and TM (p) polarization. For details see [10] and [11]. As explained in the main text, the effective magnetic permeability has an effect mainly in the TE polarization characteristics of planar metamaterials, similar to the effective dielectric permittivity pertaining to TM properties. As can be seen from the last term in Eq. (1), in order for the TE polarization to contribute to the density of optical states, the TE complex reflection coefficient must deviate from the value of $-1$.

We perform analytical transfer matrix calculations and reflection pole method calculations, together with finite element simulations investigating the TE properties of a fifty-five layers metamaterial consisting of silver and a dielectric material with refractive index $n_{diel}$, in the lossless-limit, to simplify our analysis (similar to the one discussed in Figs. 4d, e of the main text). All finite element simulations (including Figs. 1, 4d, e of the main text and Supplementary Figs. 5a, 6a) were performed with the commercial package Comsol Multiphysics. We set the refractive index of the surrounding medium to be $n_{sur} = 1.55$, similar to Figs. 4d, e of the main text. Supplementary Figs. 5, 6 show two cases: $n_{diel} = 1.5$ and $n_{diel} = 4$, respectively. The latter is the same as the case discussed in

Fig. 4e of the main text.

In the case of $n_{\text{diel}} = 1.5$ , as seen in Supplementary Fig. 5a, no TE electromagnetic states couple to the multilayer metamaterial and its response is reflective. This is confirmed with analytical transfer matrix calculations of TE reflectance (Supplementary Fig. 5d), which is almost unity, while absorption is negligible (Supplementary Fig. 5e). Furthermore, as can be seen in Supplementary Fig. 5c, $\text{Re}(r^s)$ barely deviates from the value of $-1$, indicating absence of TE-polarization electromagnetic states, based on Eq. (1). With use of the arguments explained above regarding the reflection pole method, we present the parameter $\partial m_{11}(\lambda) / \partial \lambda$ in Supplementary Figs. 5f and g for radiation (bulk) and bounded (surface) modes, respectively. No peaks are observed, confirming the absence of any TE states supported in the metamaterial in this frequency regime for the considered wavenumbers. The wavenumbers accounted for in Supplementary Figs. 5f, g are chosen based on the Gaussian beam in the finite element simulation shown in Supplementary Fig. 5a.

Furthermore, by homogenizing the layered metamaterial through [5], we obtain negative dielectric permittivity $\varepsilon_{\text{o}}$ while the magnetic permeabilities along both coordinate directions $\mu_{\text{o}}$ and $\mu_{\text{e}}$ remain positive (Supplementary Fig. 5b). For these values of $\varepsilon_{\text{o}}$ , $\mu_{\text{o}}$ and $\mu_{\text{e}}$ , the dispersion equation for bulk propagating modes $\frac{k_{\text{x}}^2 + k_{\text{y}}^2}{\varepsilon_{\text{o}}\mu_{\text{e}}} + \frac{k_{\text{z}}^2}{\varepsilon_{\text{o}}\mu_{\text{o}}} = \frac{\omega^2}{c^2}$ has no solutions, confirming, again, the absence of any states at in this frequency regime.

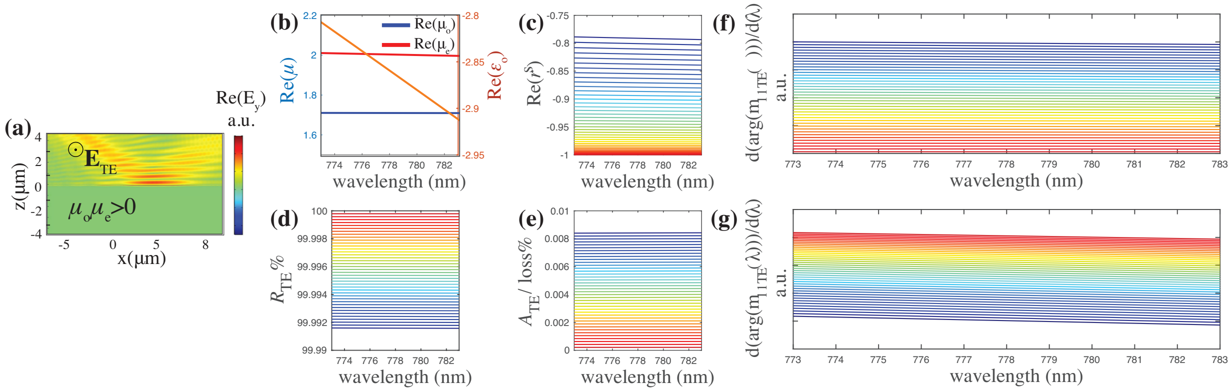


Supplementary Figure 5: **Reflection pole method and finite element simulations for $n_{\text{diel}}$=1.5.** (a) Simulation results for a fifty-five layers dielectric $n_{\text{diel}}$: 50 nm/Ag: 20 nm multilayer metamaterial at 780 nm for TE polarization, $n_{\text{diel}}$=1.5, the surrounding medium has index $n_{\text{sur}}$=1.55. (b) Effective parameters for the metamaterial in (a). (c) Re($r^s$) barely deviates from the value of -1. (d) TE polarization reflectance=$|r^s|^2$. (e) TE polarization absorption. (f) reflection pole method for radiation (bulk) modes, (g) reflection pole method for bound (surface) modes. Note 1: (c), (d), (e), (f), (g) are transfer matrix analytical multilayer calculations for the multilayer described in (a). Note 2: For (c), (d), (e), (f): Colours: from blue to red: $k_{//}$ from 1.45$k_{\text{o}}$ (blue) to 1.55$k_{\text{o}}$ (red)-corresponding to the wavenumbers in the simulation of (a). Note 3: For (g): Colours: from blue to red: $k_{//}$ from 1.55$k_{\text{o}}$ (blue) to 2$k_{\text{o}}$ (red).

In contrast, increasing the dielectric index to $n_{diel} = 4$ leads to a drastically different metamaterial response, as shown in Supplementary Fig. 6a. The metamaterial absorbs most of the incident field, as confirmed through analytical simulations showing enhanced TE absorption in Supplementary Fig. 6e, while the TE reflectance vanishes at resonant wavelengths (Supplementary Fig.

6d). As seen in Supplementary Fig. 6c, now $Re(r^s)$ is drastically different from unity, indicating a TE contribution to the density of states, based on Eq. (1). The density of states enhancement is already obvious by the enhanced absorption, however we also show it explicitly in Supplementary Fig. 6f; the reflection pole peaks represent states and their number is equal to the number of bulk eigenmodes (or optical states) for the considered wavenumbers and wavelengths. The number of TE bound (surface) states is also enhanced, as peaks are also observed for wavenumbers $k_{//} / k_o > n_{sur}$ (Supplementary Fig. 6g). This response is also interpreted in the effective homogeneous slab picture (with effective parameters $\varepsilon_o$, $\mu_o$ and $\mu_e$); the TE response of the metamaterial is now hyperbolic as $\mu_o \mu_e < 0$ (Supplementary Fig. 6b). Equivalently to the consensus that HMMs with $\varepsilon_o \varepsilon_e < 0$ support an enhanced density of TM states [12-13], we show a similar response for TE polarization due to $\mu_o \mu_e < 0$.

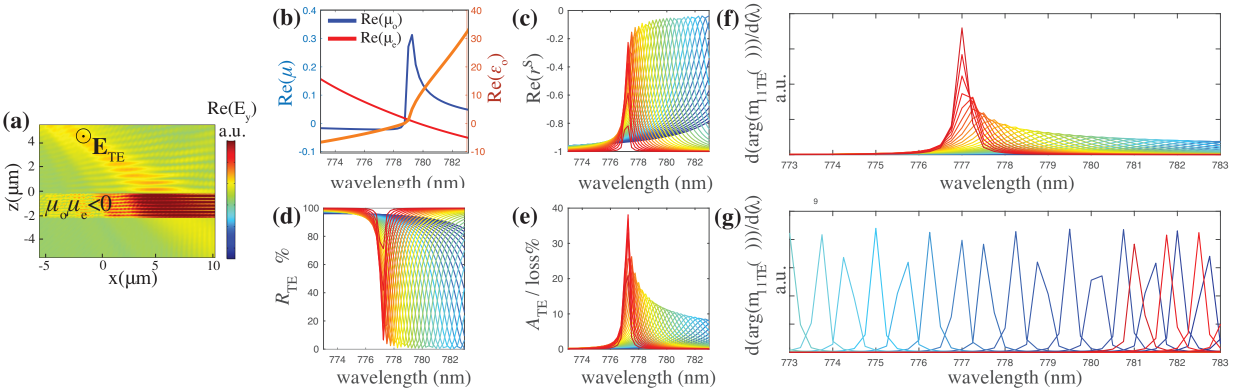


Supplementary Figure 6: **Reflection pole method and finite element simulations for $n_{diel}$=4.** (a) Simulation results for a fifty-five layers dielectric $n_{diel}$: 50 nm/Ag: 20 nm multilayer metamaterial at 780 nm for TE polarization, $n_{diel}$=4, the surrounding medium has index $n_{sur}$=1.55. (b) Effective parameters for the metamaterial in (a). (c) Re($r^s$) deviates from the value of -1 indicating enhanced density of states. (d) TE polarization reflectance=$|r^s|^2$. (e) TE polarization absorption. (f) reflection pole method for radiation (bulk) modes, (g) reflection pole method for bound (surface) modes. Note 1: (c), (d), (e), (f), (g) are transfer matrix multilayer calculations for the multilayer described in (a). Note 2: For (c), (d), (e), (f): Colours: from blue to red: $k_{//}$ from 1.45$k_o$ (blue) to 1.55$k_o$ (red)-corresponding to the wavenumbers in the simulation of (a). Note 3: For (g): Colours: from blue to red: $k_{//}$ from 1.55$k_o$ (blue) to 2$k_o$ (red).

**Supplementary Note 4:** Surface waves

In the main text Fig. 5 we examine surface-localized bound modes. To detect them, we use the reflection pole method [7] for multilayers, as described in Supplementary Note 3, and we set $k_{//} / k_o > n_{sur}$ to ensure exponential decay outside the multilayer structure. This condition alone, indicated in Supplementary Fig. 7 with C1, does not suffice for a state to be surface localized. One must impose an additional condition of the frequency of the mode to be lie inside the band gap of the metamaterial, in order to ensure decay in the metamaterial volume and therefore surface confined propagation. We show this condition in Supplementary Fig. 7 with C2. This means that the component of the wavevector normal to the interfaces has to satisfy $\mathrm{Im}(k_{zeff}) >> \mathrm{Re}(k_{zeff})$. Thus, the surface waves discussed in Fig. 5 of the main text decay away from both sides of the multilayer metamaterials interface.

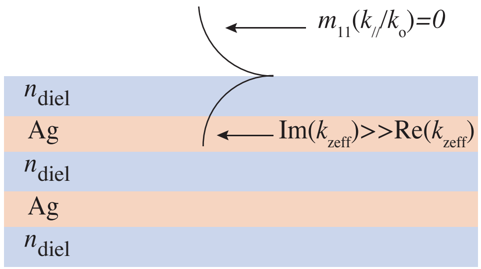


Supplementary Figure 7: **Our approach for surface wave computations:** Condition C1 ensures the eigenmode existence ($k_{//}/k_o > n_{sur}$), whereas condition C2 ensures surface wave characteristics ($Im(k_{zeff}) >> Re(k_{zeff})$), hence, interface-bound propagation.

For the structures discussed in the main text, we display in Supplementary Figs. 8, 9 numerical results for the propagation decay length $L$ and penetration depth $t$, respectively.

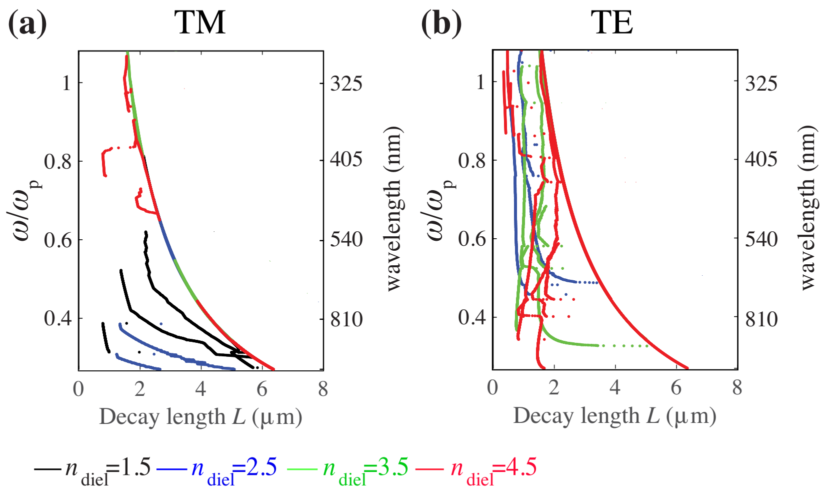


Supplementary Figure 8: **Decay length of surface waves.** Complementary to Fig. 5 of the main text. (a) TM and (b) TE surface wave decay length L=1/2Im($k_{//}$) for a 5 layers dielectric $n_{diel}$: 55nm/Ag: 25nm metamaterial.

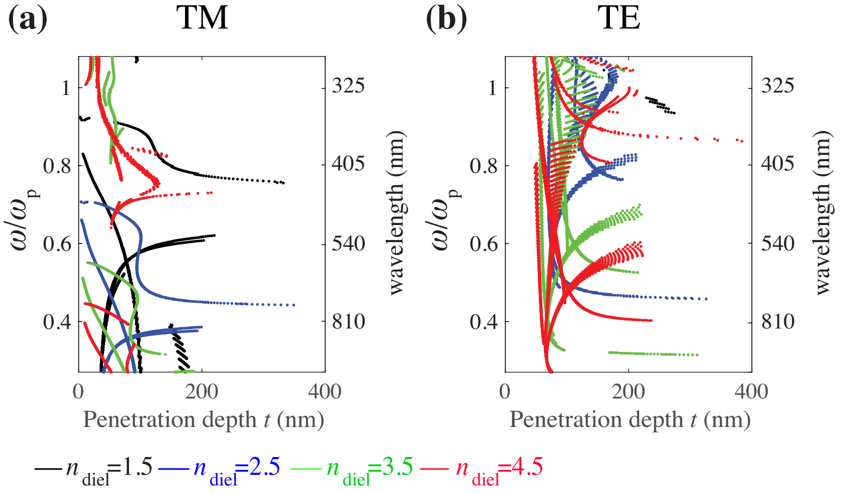


Supplementary Figure 9: **Penetration depth of surface waves.** Complementary to Fig. 5 of the main text. (a) TM and (b) TE surface wave penetration depth t=1/2Im($k_z$) for a 5 layers dielectric $n_{diel}$: 55 nm/Ag: 25 nm metamaterial.

It is well known that at the interface between a dielectric and a metal with negative dielectric permittivity, one can excite TM-polarized surface plasmonic waves, with dispersion $k=\frac{\omega}{c}\sqrt{\frac{\varepsilon_1\varepsilon_2(\omega)}{\varepsilon_1+\varepsilon_2(\omega)}}$, where $\varepsilon_1$ stands for the permittivity of the dielectric medium and $\varepsilon_2(\omega)<0$ is the dielectric permittivity of the metal [14-15]. Due to Maxwell's equations duality between TM and TE polarizations, with the exchange of dielectric permittivity with magnetic permeability, the same holds for a TE-polarized magnetic plasmon mode. Specifically, at the interface between a magnetic material with $\mu_2(\omega)<0$ and another medium with permeability $\mu_1$ a TE surface wave exists with dispersion $k=\frac{\omega}{c}\sqrt{\frac{\mu_1\mu_2(\omega)}{\mu_1+\mu_2(\omega)}}$. For a magnetically and electrically anisotropic material with parameters $\varepsilon_{\text{eff}}=\text{diag}\{\varepsilon_\text{o},\varepsilon_\text{o},\varepsilon_\text{e}\}$ and $\mu_{\text{eff}}=\text{diag}\{\mu_\text{o},\mu_\text{o},\mu_\text{e}\}$, the surface plasmon equations require modification. By solving the boundary condition problem between a semi-infinite uniaxial material and air, one obtains $\omega=kc\sqrt{\frac{1-\varepsilon_\text{o}\varepsilon_\text{e}}{\varepsilon_\text{e}(\mu_\text{o}-\varepsilon_\text{o})}}$ for TM polarization. Equivalently, for TE polarization, the surface wave dispersion is: $\omega=kc\sqrt{\frac{1-\mu_\text{o}\mu_\text{e}}{\mu_\text{e}(\varepsilon_\text{o}-\mu_\text{o})}}$. Based on these equations, the fact that the dispersion curves displayed in Figs. 5a, b of the main text, pertaining to TM and TE polarization respectively, exhibit different frequency dependence can be understood from the frequency dependence of the effective parameters $\varepsilon_\text{o}(\omega)$, $\varepsilon_\text{e}(\omega)$, $\mu_\text{o}(\omega)$ and $\mu_\text{e}(\omega)$; as shown in Figs. 3a-d of the main text, the ordinary and extraordinary permeabilities $\mu_\text{o}(\omega)$ and $\mu_\text{e}(\omega)$ exhibit very different frequency dispersion from $\varepsilon_\text{o}(\omega)$ and $\varepsilon_\text{e}(\omega)$.

**Supplementary Note 5:** The $\mu$=1 assumption in an isofrequency diagram

As discussed in Fig. 6 of the main text, one way of assuring the validity of our model, based on which multilayer metamaterials are described with an effective permittivity tensor and an effective permeability tensor, is to perform an impedance-matching sanity check for normal incidence illumination ($k_{//}=0$). Here, we extend this analysis to larger wavenumbers ($k_{//}>k_\text{o}$) and compare our model ($\varepsilon_\text{o},\varepsilon_\text{e},\mu_{\text{o},}\mu_\text{e}$) with the traditional EMA. We do so by re-assessing the TE bulk-modes discussed in Figs. 5a, b, c of our main text, not only in the ($\varepsilon_\text{o},\varepsilon_\text{e},\mu_{\text{o},}\mu_\text{e}$) picture, but also in the nonmagnetic ($\varepsilon_{\text{o},\mu=1},\varepsilon_{\text{e},\mu=1}$) picture, by setting a priori the magnetic permeability to unity.

As shown in Fig. 6c of the main text, setting a priori the magnetic response to unity yields similar results to the Maxwell Garnett approach. We take as an example the $n_{\text{diel}}=2.5$ /silver metamaterial, same as the one discussed in Figs. 4a-c of the main text with the blue curves. The TE reflectance (Supplementary Fig. 10a) exhibits very pronounced angle (or wavenumber) dependence, which can be understood in terms of an effective anisotropic magnetic response, since the dispersion of TE propagating modes is given by $\frac{k_\text{x}^2+k_\text{y}^2}{\varepsilon_\text{o}\mu_\text{e}}+\frac{k_\text{z}^2}{\varepsilon_\text{o}\mu_\text{o}}=\frac{\omega^2}{c^2}$. This is in agreement with Supplementary Fig.

10b; the open, anisotropic dispersion agrees well with the angle dependence in reflectance $R_{\mathrm{TE}}$. Contrary to this, the non-magnetic model ($\varepsilon_{\text{non-mag.}}, \mu = 1$) (with Supplementary Fig. 10c) yields a circular isofrequency contour (for $k_{//} / k_{\mathrm{o}} \leq 1$). Circular isofrequency diagrams, however, imply an isotropic dispersion, which is not in agreement with the angle-dependence of $R_{\mathrm{TE}}$. We note that $R_{\mathrm{TE}}$ was calculated using the physical dielectric/metal geometry as explained in the main text. We also note that we take into account spatial dispersion effects when calculating the isofrequency diagrams.

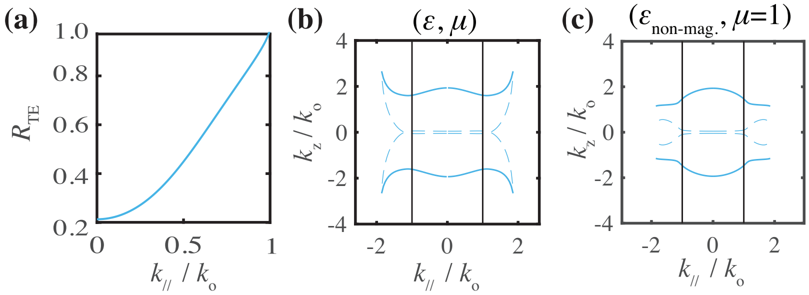


**Supplementary Figure 10: $\mu_{eff} = 1$ additional sanity check, complementary to Figs. 4a, c of the main text.** (a) TE polarization reflectance for a 5 layers dielectric $n_{\text{diel}}$=2.5, 55nm/Ag: 25nm metamaterial. Isofrequency diagrams for TE polarization at 500 nm when: (b) magnetic effects accounted for and (c) neglecting magnetic effects. Solid lines: real parts, dashed lines: imaginary parts.

## Supplementary References


[1] Landau, L., Lifshitz, E. & Pitaevskii, L. P. Electrodynamics of continuous media 2nd edition, 8 (Pergamon Press, 1984).

[2] Mirmoosa, M. S., Kosulnikov, S. Y. & Simovski, C. R. Magnetic hyperbolic metamaterial of high-index nanowires. *Phys. Rev. B* 94, 075138(1)–075138(8) (2016).

[3] Smith, D. R. & Pendry, J. B. Homogenization of metamaterials by field averaging (invited paper). *J. Opt. Soc. Am. B* 23, 391–403 (2006).

[4] Penciu, R. S. et al. Multi-gap individual and coupled split-ring resonator structures. Opt. Express 16, 18131– 18144 (2008).

[5] Papadakis, G. T., Yeh, P. & Atwater, H. A. Retrieval of material parameters for uniaxial metamaterials. *Phys. Rev. B* 91, 155406(1)–155406(12) (2015).

[6] Agranovich, V. & Kravtsov, V. Notes on crystal optics of superlattices. *Solid State Communications* 55, 85 – 90 (1985).

[7] Anemogiannis, E., Glytsis, E. N. & Gaylord, T. K. Determination of guided and leaky modes in lossless and lossy planar multilayer optical waveguides: reflection pole method and wavevector density method. *Journal of Lightwave Technology* 17, 929–941 (1999).

[8] Yeh, P. Optical Waves in Layered Media 2nd edition (Wiley-Interscience, 2005).

[9] Chen, C., Berini, P., Feng, D., Tanev, S. & Tzolov, V. P. Efficient and accurate numerical analysis of multilayer planar optical waveguides in lossy anisotropic media. *Opt. Express* **7**, 260-272 (2000)

[10] Cortes, C. L., Newman, W., Molesky, S. & Jacob, Z. Quantum nanophotonics using hyperbolic metamaterials. *Journal of Optics* **14**, 063001 (2012).

[11] Cortes, C. L. & Jacob, Z. Photonic analog of a van Hove singularity in metamaterials. *Physical Review B* **88**, 045407 (2013).

[12] Jacob, Z. *et al*. Engineering photonic density of states using metamaterials. *Applied Physics B* **100**, 215-218, (2010).
[13] Krishnamoorthy, H. N. S., Jacob, Z., Narimanov, E., Kretzschmar, I. & Menon, V. M. Topological transitions in metamaterials. Science 336, 205–209 (2012).
[14] Pitarke, J. M., Silkin, V. M., Chulkov, E. V. & Echenique, P. M. Theory of surface plasmons and surface-plasmon polaritons. *Reports on Progress in Physics* **70**, 1 (2007).
[15] Economou, E. N. Surface Plasmons in Thin Films. *Physical Review* **182**, 539-554 (1969).
